# Decoding Picky Eating in Children: A Temporary Phase or a Hidden Health Concern?

**DOI:** 10.3390/nu17243884

**Published:** 2025-12-12

**Authors:** Dorina Pjetraj, Amarildo Pjetraj, Dalia Sayed, Michele Severini, Ludovica Falcioni, Lucia Emanuela Svarca, Simona Gatti, Maria Elena Lionetti

**Affiliations:** 1 Department of Pediatric Emergency, Salesi Children Hospital, AOU delle Marche, 60123 Ancona, Italy; 2 Faculty of Education, Health & Human Sciences, School of Health Sciences, University of Greenwich, London SE9 2UG, UK; pjetraj.amarildo@gmail.com; 3 Department of Neonatology and Neonatal Intensive Care, University Hospital Lewisham, London SE13 6LH, UK; daliaamsayed@gmail.com; 4 Department of Eating Disorders in Childhood and Adolescence with Comorbid Psychiatric Conditions, AOU delle Marche, 60123 Ancona, Italy; michele.severini@ospedaliriuniti.marche.it (M.S.); ludovica.falcioni@ospedaliriuniti.marche.it (L.F.); luciaemanuela.svarca@ospedaliriuniti.marche.it (L.E.S.); 5 Department of Pediatrics, Marche Polytechnic University, 60123 Ancona, Italy; s.gatti@staff.univpm.it (S.G.); m.e.lionetti@staff.univpm.it (M.E.L.)

**Keywords:** picky eating, food selectivity, feeding disorders, ARFID, child nutrition, behavioral interventions, pediatric feeding

## Abstract

**Background**: Picky eating (PE), also termed food selectivity, is one of the most common feeding concerns in childhood. Although often a transient developmental stage, persistent or severe selectivity may lead to nutritional deficiencies, growth impairment, and psychosocial consequences. **Methods**: This narrative review is based on literature searches conducted in April 2025 across PubMed, Web of Science, Embase, Medline, and Google Scholar. Articles published between 2015 and 2025 were included if they addressed the epidemiology, etiology, assessment, or management of PE in children aged 0–18 years. Additional seminal references predating this period were also considered. **Results**: Prevalence estimates of PE vary widely (13–50%), with peak incidence between ages two and six. Contributing factors include genetic predisposition, sensory sensitivities, temperament, family feeding practices, environmental influences, and adverse feeding experiences. Distinction from avoidant/restrictive food intake disorder (ARFID) and pediatric feeding disorder (PFD) is essential, as these conditions carry greater risk of nutritional and psychosocial impairment. Assessment relies on caregiver-report instruments, clinical observation, growth monitoring, and targeted nutritional evaluation. Effective management integrates parental education, responsive feeding strategies, repeated exposure to novel foods, and, when indicated, nutritional supplementation or referral to multidisciplinary teams. Sensory-based therapies, behavioral interventions, and psychoeducational programs show particular benefit in persistent cases. **Conclusions**: While most children outgrow PE without adverse outcomes, a subset remains at risk of long-term nutritional compromise and psychosocial difficulties. Early recognition, family-centered guidance, and evidence-based interventions are essential. Future research should refine diagnostic criteria, develop culturally sensitive assessment tools, and evaluate innovative therapies to improve outcomes.

## 1. Introduction

Picky eating (PE), also referred to as food selectivity, fussy, faddy or choosy eating, describes a spectrum of behaviors characterized by unwillingness to eat certain foods, preference for a narrow diet, and aversion to unfamiliar tastes or textures. While some level of pickiness is typical during early childhood, persistent or extreme cases may lead to nutritional imbalances and warrant clinical attention.

Concerns about PE often originate from caregivers rather than from health consequences. Parental anxiety may stem from unrealistic expectations about food intake and nutrition. This underscores the importance of distinguishing between developmentally appropriate behaviors and feeding disorders requiring intervention.

In recent years, formal diagnostic frameworks have broadened the understanding of pediatric feeding problems. The Diagnostic and Statistical Manual of Mental Disorders, Fifth Edition (DSM-5) includes several feeding and eating disturbances relevant to childhood, such as Pica, and Rumination Disorder and Avoidant/Restrictive Food Intake Disorder (ARFID)—a condition characterized by restrictive food intake associated with nutritional deficiency, weight loss, dependence on supplements, or marked psychosocial impairment. These classifications highlight the complex interplay between sensory sensitivities, behavioral patterns, medical conditions, and developmental factors that influence children’s feeding [1]. Within this context, the present review synthesizes current evidence on the epidemiology, etiology, assessment, and management of picky eating in pediatric populations. The goal is to clarify the distinction between transient developmental phases of selectivity and clinically significant feeding disturbances, while identifying areas in need of greater standardization and research.

## 2. Materials and Methods

This narrative review was conducted through a systematic and transparent literature search performed in April 2025 across five major academic databases: PubMed, Embase, Web of Science, Medline, and Google Scholar. The search strategy incorporated a comprehensive set of Medical Subject Headings (MeSH) and free-text keywords, including: “picky eating”, “food selectivity”, “feeding difficulties”, “food neophobia”, “Avoidant/Restrictive Food Intake Disorder”, “pediatric feeding disorder”, “child nutrition”, and “feeding behavior”. Boolean operators (e.g., AND, OR) were used to refine and combine search terms.

The search targeted articles published from January 2015 to April 2025. Seminal or foundational studies predating this timeframe were also included when clinically or conceptually relevant. Eligible studies met the following inclusion criteria: (1) focused on children aged 0–18 years; (2) addressed the etiology, assessment, diagnosis, prevalence, or management of picky eating or related feeding disorders; and (3) published in English. Exclusion criteria included studies involving adults only, articles lacking primary data (e.g., opinion pieces), and publications not directly addressing feeding behavior.

Screening was performed in two stages: first by title and abstract, and then by full-text review. Two independent reviewers completed the screening process, with disagreements resolved through discussion and, when necessary, consultation with a senior reviewer. The search initially identified 1243 records, of which 842 remained after deduplication. Following title and abstract screening, 111 studies were included. An additional 29 studies were identified through manual searches of bibliographies and reference lists of key articles, resulting in a total of 140 studies included in the final narrative synthesis.

Given the heterogeneity of study designs, populations, and measurement tools across the literature, a narrative synthesis approach was chosen. This method allowed integration of findings from observational studies, cohort data, clinical reports, and intervention trials. Methodological variability, including reliance on parental-report instruments, cultural differences, and sampling biases, was acknowledged and considered in interpreting the results. The limitations inherent to narrative reviews—such as potential selection bias and lack of meta-analytic quantification—were mitigated through transparent reporting of search procedures and explicit justification for study inclusion.

## 3. Definition

The interpretation of “PE” in the pediatric population is challenged by a lack of unified terminology across the literature. Studies alternately refer to food neophobia, selective eating, sensory-based avoidance, or restrictive feeding behaviors, often without clear operational distinctions. These discrepancies contribute to the wide prevalence range reported globally, as different constructs capture overlapping but not identical behaviors. Additionally, prevalence estimates vary depending on whether behaviors are assessed through parental perception, structured clinical tools, or observational methods. Cultural influences—including dietary norms, accepted feeding practices, and levels of urbanization—further complicate cross-study comparability. Recognizing this variability is essential for understanding the true epidemiological burden of picky eating and underscores the need for standardized definitions [2]. Dovey and colleagues’ foundational definition portrays picky eaters as those who reject a substantial proportion of available foods—embracing both unfamiliar items and a subset of familiar foods—thereby limiting dietary variety; this concept explicitly acknowledges components of food neophobia and texture-based avoidance [3]. Building on this, Mascola et al. and Taylor et al. operationalize pickiness in terms of the number of foods a child will accept, using food-item checklists or count-based scales to quantify dietary restriction [4,5,6]. Others emphasize parental perceptions, defining PE by its impact: eating behaviors that “disrupt daily routines to an extent that is problematic to the parent, child, or parent–child relationship” [7].

Definition is further complicated by the subjective lens through which eating behaviors are viewed by caregivers. Up to half of parents label their young child a “picky eater” at some point, reflecting cultural norms, familial expectations, and individual tolerance for mealtime conflict [8,9]. Indeed, families of picky eaters often report heightened stress around meals, inconsistent social support, and the adoption of varied coping strategies—from insisting on a single bite to preparing entirely separate dishes or permitting meal skipping—which can inadvertently reinforce selectivity [10,11].

In practice, this definitional heterogeneity has spurred the development of over a dozen assessment instruments—from broad screening tools like the Children’s Eating Behaviour Questionnaire to focused scales querying specific feeding behaviors—each capturing different facets of selectivity [5,12].

Distinguishing PE from related constructs is essential. Food neophobia—characterized strictly by the rejection of novel foods—affects 40–60% of children and reflects an adaptive wariness of unknown items [9,13,14]. In contrast, PE encompasses reluctance toward both familiar and unfamiliar foods, often driven by sensory, behavioral, and psychosocial factors [15]. Similarly, “fussy”, “choosy”, and “selective” eating are used interchangeably in the literature but may connote subtle differences in parental framing or measurement focus [4,5].

To achieve greater clinical clarity, a recent consensus panel delineated three severity tiers [16]:

- Mild PE: Suboptimal dietary variety or quality without evidence of nutrient deficiencies or growth faltering.
- Moderate PE: Similar restrictive patterns accompanied by laboratory markers of under- or overnutrition or micronutrient insufficiency.
- Severe PE: Extreme food rejection leading to significant nutritional deficiencies, impaired growth trajectories, or marked psychosocial distress.

This framework bridges the gap between normative, developmentally typical PE—which peaks between two and six years of age and often remits spontaneously—and more entrenched feeding disorders such as Avoidant/Restrictive Food Intake Disorder (ARFID) categorized in DSM-5, where restriction stems from sensory sensitivities, fear of adverse consequences, or indifference to eating [17].

## 4. Epidemiology and Prevalence

PE affects a substantial proportion of young children and ranks among the most frequent concerns voiced by caregivers during pediatric visits. Reported prevalence estimates vary widely—from approximately 13% to over 50%—largely depending on the child’s age, the operational definition employed, and the assessment instrument used [5,18,19,20,21]. A comprehensive meta-analysis, pooling data across diverse studies, estimated the overall prevalence of PE in childhood at around 22%, highlighting how methodological heterogeneity shapes our understanding of this behavior [22].

Epidemiological investigations consistently show that PE emerges in early toddlerhood, with prevalence peaking between two and six years of age. This developmental window coincides with the acquisition of greater autonomy, heightened sensory awareness, and the adaptive emergence of neophobic responses, thought to have evolutionary origins as a protective mechanism against ingesting potentially harmful substances [23].

Large-scale longitudinal cohorts have provided valuable insights into the natural history of PE. The Avon Longitudinal Study of Parents and Children (ALSPAC), which enrolled over 14,000 pregnant women in the early 1990s and has followed their offspring through multiple developmental stages, assessed PE at 24, 38, 54, and 65 months via parental reporting. ALSPAC data revealed a modest prevalence of PE—ranging from 9.7% to 14.7%—with the highest rate at 38 months, underscoring a peak in mid-toddlerhood [24].

A complementary U.S.-based longitudinal study by Fernandez et al. followed 317 low-income mother–child dyads from ages four to nine years, administering the CEBQ food-fussiness subscale at five intervals and employing latent class analysis to delineate three stable picky-eating trajectories. They identified persistently low (29%), medium (57%), and high (14%) levels of pickiness, providing robust evidence for the long-term stability and distinct subgroups of PE within socioeconomically vulnerable populations [25].

Additional cohort studies furnish converging evidence on age-related trends. For instance, Theresa et al. reported a prevalence of 26.5% at 18 months, declining to 13.2% by six years, signaling a natural remission in many children [26]. Likewise, Taylor et al. documented a pronounced peak in PE at 38 months in a large community sample, reinforcing the notion of a critical window in mid-toddlerhood [5]. More recent data from Bourne et al. indicate that while transient PE affects some 23.3% of children in early childhood, only about 3.7% exhibit persistent pickiness into late childhood; the majority remit by adolescence, though a small subset remains at risk of longer-term feeding difficulties [21]. By pre-adolescence, the combined prevalence of PE and food neophobia has been estimated at 34% and 14%, respectively [27]. Families can find reassurance in data such as that of Diamantis et al. [28], who observed that feeding difficulties often improve gradually as children enter the school years.

Importantly, pickiness shows no consistent sex bias, affecting girls and boys equally [18,24,29], suggesting that biological susceptibility to PE is not sex-specific.

Cross-cultural investigations confirm that PE is a global phenomenon. In Singapore, nearly half (49.6%) of children aged 1–10 years were reported as picky by their caregivers [30]. In China, prevalence estimates range from 36% among 24–35-month-olds to 59.3% in 7–12-year-olds; Taiwan reports a 62% rate among similar age groups, while 35.6% of preschoolers in Kuwait meet picky eater criteria [31,32,33,34]. These international data illuminate the role of cultural norms, dietary practices, and parental expectations in shaping both the perception and expression of PE.

## 5. Developmental Considerations and Feeding Milestones

Feeding is one of the earliest and most complex developmental tasks a child undertakes, requiring the integration of motor skills, sensory processing, emotional regulation, and social interaction. Historically, feeding behaviors have not always been systematically included in standardized developmental surveillance tools. However, recent updates to the Centers for Disease Control and Prevention (CDC) developmental milestone checklists, guided by the American Academy of Pediatrics (AAP) [35], provide an opportunity to reframe how feeding-related behaviors are monitored and addressed during early childhood development.

One of the most significant changes in the revised CDC developmental surveillance tools is the shift from reporting median age milestones to those achieved by at least 75% of children at a given age. This approach aims to support timely identification of developmental delays and avoid the historically common “wait-and-see” response, which has contributed to delays in intervention, particularly in domains such as language, social-emotional development, and feeding behaviors [35].

Although feeding itself is not categorized as a distinct developmental domain in the CDC checklist revisions, related behaviors span multiple areas, notably motor, social-emotional, and cognitive domains. These behaviors are critical components of early developmental progression and often serve as initial indicators of neurodevelopmental integrity [35].

### 5.1. Infancy (0–6 Months)

In the early months of life, feeding is largely reflexive. Rooting, sucking, and swallowing are essential motor milestones. Infants gradually develop the ability to coordinate sucking with breathing and swallowing, a skill foundational for both nutritional intake and later speech development. During this period, feeding also supports social-emotional bonding, as infants begin to recognize caregivers and respond to their voices and touch.

### 5.2. 6–12 Months

The introduction of solid foods marks a pivotal transition in feeding development. During this time, infants begin to develop oral-motor control necessary for chewing and manipulating food. Concurrently, they exhibit intentional actions such as reaching for food, holding a bottle, or mouthing objects—early signs of self-feeding and fine motor coordination. Socially, they start to demonstrate food preferences, facial expressions in response to taste, and anticipatory behaviors when mealtimes approach.

### 5.3. 12–24 Months

Between one and two years of age, toddlers increasingly assert independence in feeding. Milestones during this period include using utensils with variable success, drinking from open cups, and participating in mealtime routines. Social-emotional growth is evident as children engage in shared meals, observe and imitate the eating behaviors of others, and begin to express likes and dislikes verbally or through gestures. Cognitive development contributes to understanding simple instructions related to mealtime and participating in pretend play scenarios that mimic eating and cooking behaviors.

### 5.4. 2–5 Years

Preschool-aged children further refine self-feeding skills and participate more fully in the social aspects of eating. They follow structured mealtime routines, demonstrate table manners, and engage in conversation during meals. This age range also marks the emergence of imaginative play involving food, such as pretending to be a chef or hosting a tea party, indicating a growing capacity for symbolic thinking and social interaction. Notably, persistent feeding difficulties at this stage may signal broader developmental delays or atypical sensory processing and warrant closer surveillance. PE often emerges during this transition period as children assert their autonomy and express preferences for certain foods while rejecting others.

Taste is a critical neuronal sensation that allows individuals to evaluate the content of food, thereby influencing food selection and overall dietary intake. Beyond guiding preference, taste plays a fundamental role in determining the nutritional or harmful properties of foods, enabling the identification and avoidance of potentially toxic substances. During early childhood, taste-related behaviors evolve through distinct developmental phases. In the first year of life, a stage of neophilia is typically observed, during which infants are open to new flavors and textures, particularly around the time of weaning and the introduction of solid foods. Between one and three years of age, this is subsequently followed by a phase of “neophobia”, characterized by reluctance to try unfamiliar foods, which is thought to reflect an evolutionary protective mechanism against the ingestion of toxins. Importantly, frequent or persistent feeding problems that extend into the fifth year of life may serve as early indicators of developmental delay, highlighting the value of targeted screening in this population [36,37].

## 6. ARFID and Other Feeding Disorders

Since the beginning of the 21st century, the understanding of Feeding and Eating Disorders in children has significantly evolved. The DSM-5 classification, released in 2013, established new diagnostic categories applicable across all age groups. These include Avoidant/Restrictive Food Intake Disorder (ARFID), Binge-eating Disorder, Other Specified Feeding Disorders (FED), and Unspecified Feeding Disorder (UFED), supplementing the previously recognized conditions of Anorexia Nervosa, Bulimia Nervosa, Pica, and Rumination Disorder [38]. Due to these relatively recent classifications, prevalence data for ARFID and UFED in the general pediatric population remain scarce. While Bulimia Nervosa and Binge Eating Disorder are more commonly observed in adolescents, Anorexia Nervosa (AN), ARFID, pica, and rumination have been documented in both children and adolescents [39,40]. A recent study examining the distribution of Feeding and Eating Disorders in children under 12 years of age indicated that ARFID was the most prevalent, followed by Unspecified Feeding and Eating Disorder and Anorexia Nervosa. These findings highlight the clinical significance of ARFID within pediatric populations [41].

ARFID is a distinct feeding and eating disorder characterized by significant nutritional deficits, weight loss, psychosocial impairment, and dependence on supplements. Unlike anorexia nervosa or bulimia, ARFID is not driven by concerns about body weight or shape. It typically presents in childhood or adolescence and may resemble extreme PE, but with more severe consequences. ARFID is associated with limited dietary repertoire, sensory sensitivity, fear of adverse consequences, and lack of interest in eating. Prevalence estimates range from 3 to 5% in the general pediatric population, with higher rates among those with feeding difficulties or eating disorders. ARFID frequently coexists with conditions like autism spectrum disorder, anxiety, and sensory processing issues. Up to 20–40% of children with autism spectrum disorder (ASD) may exhibit ARFID-like behaviors. Diagnosis of ARFID is based on meeting at least one of the DSM-5 criteria, which include (1) significant weight loss or failure to achieve expected weight gain, (2) significant nutritional deficiency, (3) dependence on enteral feeding or oral nutritional supplements, and (4) marked interference with psychosocial functioning [38]. The diagnosis involves a comprehensive assessment process, evaluating growth parameters, nutrient intake, and behavioral observations. Diagnostic tools such as the PARDI (Pica, ARFID, and Rumination Disorder Interview) and the Nine Item ARFID Screen (NIAS) [42] are increasingly used in research and clinical practice. Recent literature describes three main ARFID presentations: sensory-based avoidance (e.g., refuses foods with certain textures or colors), fear-based avoidance (e.g., post-choking or emesis trauma), and low appetite/interest in eating (e.g., eats very small amounts, shows little hunger) [43]. These subtypes can co-occur and are not mutually exclusive. Given the high co-morbidity between ARFID and ASD, those diagnosed with ARFID should also be evaluated for ASD, and vice versa [44].

Children with AN were often referred to psychiatry, while those with ARFID or UFED might be diagnosed by either gastroenterologists or psychiatrists, based on their symptoms [41]. As these children often have related health and mental health issues, they warrant a thorough evaluation and care from a multidisciplinary team of specialists. Early intervention is important to lessen the effects of the disorder on their development and well-being [45].

In 2019, the World Health Organization (WHO) introduced the concept of pediatric feeding disorder (PFD), offering a more comprehensive view of typical feeding development across four areas: medical, nutritional, feeding skill, and psychosocial dysfunction. PFD is characterized by a disturbance in nutrient intake lasting a minimum of two weeks, which is incongruent with age-appropriate development [46]. This integrated definition allows for a more nuanced assessment of feeding difficulties, moving beyond a sole focus on food refusal to encompass broader functional impairments [47]. While ARFID focuses on internal and psychological factors leading to restricted intake, PFD encompasses a wider array of etiologies, including physiological and structural issues that impact feeding [48]. There is, however, a clear overlap between the two diagnostic frameworks, particularly in cases where sensory sensitivities or learned aversions contribute to significant nutritional deficiencies and functional impairments. PFD and ARFID can exhibit an interdependent relationship, with individuals potentially meeting criteria for both conditions, or presenting with each disorder in isolation (Table 1). For this reason, a recent consensus has expressed the need for a more refined distinction and understanding of their diagnostic overlap to better guide clinical practice and research efforts [48].

## 7. Contributing Factors

### 7.1. Genetic Factors

Distinguishing types of genetic evidence is key to understanding the genetics of picky eating. Most knowledge in this area stems from twin studies and parent surveys, while molecular studies—such as genome-wide association studies and sequencing—remain limited. Studies have a high heritability component to neophobia, but they also indicate that just under a quarter of the variance in neophobia can be attributed to non-shared environmental influences [49,50]. Another investigation indicated that broad-sense heritability for adult PE is 49% with dominance genetic effects at 35%, while the residual variance is attributed to unique environmental factors [51]. Genetic factors influence food choices through taste receptors, impacting perception of sweet, umami, and bitter tastes, affecting tolerance to foods like broccoli [52].

### 7.2. The Immune Mechanisms

The intricate interplay between the immune system and the gastrointestinal tract plays a pivotal role in shaping feeding behaviors, particularly in the context of food allergies and intolerances, which can significantly influence dietary preferences and aversions in children. Emerging evidence suggests that, similar to other allergy symptoms like sneezing, itching, or vomiting that counteract immediate exposure to an allergen, food aversion helps protect the host by facilitating subsequent avoidance of a toxin [53]. In two complementary mouse models of food allergy, immunological sensitization to an ingested allergen triggered a robust, allergen-specific avoidance behavior when the animals were later offered a choice between plain water and allergen-containing water. This avoidance response required typical allergy mediators such as IgE antibodies, IL-4, and mast cell activation, which lead to the release of leukotrienes. This response was also genetically determined by both the presence of mast cells and the specific mouse strain. Importantly, gut epithelial cells produced the hormone GDF15 in a manner dependent on IL-4/IgE when re-exposed to allergens. This GDF15 then signals to brainstem centers, triggering a conditioned food aversion. These findings reveal a dedicated neuroimmune circuit linking peripheral allergic responses to central learning pathways, effectively “priming” food avoidance through Pavlovian-style conditioning. Although these mechanisms have been demonstrated in animals, they may help explain food avoidance in allergic children. However, no studies have directly examined GDF15 in relation to picky eating in humans. Current evidence comes from animal models and research on nausea and conditioned aversion, and GDF15 has not yet been specifically studied in pediatric selective eating [53,54,55].

### 7.3. Psychological and Sensory Processing Issues and Temperament

Children who exhibit heightened sensory sensitivity in early childhood are at a markedly increased risk of developing persistent PE. Longitudinal data indicate that four-year-olds with elevated taste, olfactory, or tactile sensitivity are significantly more likely to meet criteria for PE by age six [18]. In particular, tactile defensiveness—aversion to certain food textures when touched or mouthed—correlates strongly with a narrowed food repertoire [56,57]. Sensory over-responsivity may drive children to reject foods based on mouthfeel alone, independent of flavor or nutritional content. Bellaïche et al. [58] examined feeding difficulties in children aged 1 to 6 years within a multidisciplinary outpatient setting. In this case–control study, researchers compared patients with pediatric feeding disorders assessed by a multidisciplinary team to a control group. Selective intake was the primary reason for appointments in the case group, and mealtimes were frequently a source of conflict for families. Deficits in oral motor skills were observed in the case group. Signs of sensory sensitivity were much more frequent among these patients than in the control group [58]. Specifically, a significant portion of the group with feeding disorders exhibited visual, olfactory, tactile, and intraoral hypersensitivity [58]. Moreover, alterations in normal stages of environmental exploration were evident, with decreased mouthing behaviors potentially indicating sensory aversions [58].

Between four and six years of life, taste sensitivity and preference evolve rapidly; thresholds for sourness and saltiness decline, sweetness sensitivity wanes, and concomitantly children develop a greater hedonic drive for sweeter foods [59]. Despite adult studies suggesting that taste is a major driving factor superior to appearance, odor, or texture, the concept of ‘sensory satisfaction’ has yet to be applied to pediatric picky eaters [60]. These shifting sensory landscapes suggest that timed interventions—matched to a child’s developmental stage—may optimize the impact of both repeated taste exposures and sensory desensitization techniques.

Although early temperament traits such as shyness, fearfulness, and negative emotionality are more prevalent in picky eaters, they do not reliably predict the continuation of pickiness into middle childhood, nor systematically shape parental feeding behaviors [18,61,62]. Together, these findings advocate for interventions that prioritize modifiable sensory experiences—through structured play, multisensory exposure, and thoughtfully designed eating environments—over attempts to alter stable temperament dimensions.

### 7.4. Family and Environmental Factors

Family and environmental contexts play a pivotal role in the development and maintenance of PE. Children classified in the high–picky-eating trajectory not only exhibited greater emotional lability and poorer self-regulation but also experienced more restrictive and demanding maternal feeding practices, highlighting the bidirectional dynamics of parent–child interactions around food [25]. Caregiver perceptions often mirror these dynamics: parents of picky eaters are more likely to report underweight status in their child [33] and to employ pressuring or restrictive strategies around unhealthy foods [63]. Indeed, inappropriate feeding interactions—such as coercive prompting, threats, and frequent nutrient supplementation—are significantly more common among picky eaters, whereas positive strategies like gentle encouragement and repeated, structured food exposures are less frequently used [31]. Children granted excessive control over feeding and allowed television during meals are more prone to picky behaviors, whereas a positive family mealtime atmosphere and established routines predict lower odds of selectivity one year later [64]. Moreover, coercive prompting styles—such as pressure to eat or modeling without choice—are inversely associated with actual intake of target foods like green beans, and parental pressure in childhood can presage disordered eating and maladaptive eating attitudes in young adulthood [65,66].

Maternal characteristics further influence selectivity. For instance, Kutbi et al. [67] stratified PE in school-aged children into tertiles using the Child Eating Behavior Questionnaire (CEBQ) and found that mothers with obesity had higher odds of extreme PE profiles. Moreover, maternal concerns about pickiness often correlate with depressive symptoms and negative perceptions of the child’s behavior [68]. Parental eating habits also matter—offspring of picky-eating parents are substantially more likely to develop similar eating patterns, with risk magnified 2.85 times when only the mother is a picky eater, 5.99 times when only the father is a picky eater, and a staggering 22.79 times when both parents are picky eaters [69].

Firstborn children—whose parents may lack prior caregiving experience—appear particularly vulnerable to developing persistent pickiness, underscoring the value of early parental guidance and skill development in feeding practices [18,61].

Socioeconomic contexts add further nuance. Picky eaters from lower-income backgrounds tend to follow the lowest weight-for-length trajectories [70], although other studies find no clear link between parental education or income and pickiness [32]. Also Brown et al. demonstrated that factors such as race/ethnicity, household food insecurity, and single-parent status do not consistently predict pickiness [71].

Given these intertwined influences, primary care providers should counsel families on the counterproductive nature of coercive feeding practices and promote responsive, supportive mealtime strategies that foster exploration and autonomy [72].

### 7.5. Early Feeding Experiences and Trauma

Infants who undergo invasive or distressing feeding procedures—such as prolonged endotracheal intubation or nasogastric tube dependence—are at heightened risk of oral aversion that can persist well beyond the neonatal period. A retrospective analysis in 2020 involving 18 children dependent on feeding tubes and exhibiting considerable oral aversion indicated that intensive, interdisciplinary feeding therapy led to significant decreases in aversive behaviors and tube reliance. However, these children initially presented with more pronounced refusal and hypersensitivity when contrasted with non-orally averse control subjects [73]. A 2022 meta-analysis further confirmed that feeding-tube placement is associated with higher levels of food refusal and oral-motor deficits than in children whose feeding difficulties resolve without tube support [74]. In a study of 372 patients, 29% experienced difficulty swallowing after extubation [75]. Furthermore, children under 25 months old were twice as likely to have swallowing problems after extubation and were also more likely to require a feeding tube upon hospital discharge [75].

By contrast, the predominant infant feeding mode—breast versus bottle—appears to exert only modest influence on later selectivity. A systematic review found that longer breastfeeding duration was generally linked to fewer parent-reported feeding problems, but effect sizes were small and often non-significant [76]. Alkazemi et al. similarly observed no association between breastfeeding versus formula feeding or the timing of solid-food introduction and the emergence of PE [32]. Taylor and Emmett’s cohort study further confirmed that neither feeding method nor complementary feeding onset reliably predicted later fussiness [12].

Further research indicated a strong correlation between early feeding behaviors and motor development outcomes at 4 to 5 years of age. Therefore, extremely preterm infants who exhibit early feeding challenges warrant identification as a high-risk group for suboptimal motor outcomes later in life, necessitating early screening for diagnosis and subsequent intervention [77].

Complementary feeding style, however, may shape early food acceptance. Baby-led weaning (BLW)—in which infants self-feed family foods rather than being spoon-fed purees—has been associated in observational studies with lower food fussiness, greater food enjoyment, and improved satiety responsiveness [78]. Białek-Dratwa and Kowalski found no increase in food neophobia risk with BLW, and a randomized trial by Taylor et al. revealed that BLW infants exhibited significantly less fussiness at 12 months than those following traditional spoon-feeding [79,80]. In the recent CORALS cohort of 1215 three- to six-year-olds, both extended breastfeeding and BLW or a mixed approach to weaning correlated with higher enjoyment of food and lower picky-eating scores [81].

Taken together, these findings suggest that while early traumatic feeding experiences can cement maladaptive oral aversions, routine variations in feeding modality exert subtler effects (Table 2). Complementary feeding approaches that encourage infant autonomy and sensory exploration—especially BLW—show promise in fostering acceptance and reducing fussiness, whereas prolonged negative associations with feeding via medical devices may require targeted desensitization and specialized tube-weaning interventions.

## 8. Clinical Assessment Tools

Effective evaluation of pediatric feeding difficulties requires a multidimensional approach that integrates caregiver reports, direct clinical observation, validated assessment tools, and the child’s nutritional and growth data. The goal is to distinguish between typical PE and more serious feeding disorders such as Avoidant/Restrictive Food Intake Disorder (ARFID), while also identifying any underlying medical, psychological, or sensory-motor factors that may contribute to the feeding challenges.

A wide range of parental-report instruments have been developed to assess various aspects of pediatric feeding behaviors, food preferences, and mealtime interactions for both clinical and research purposes, with over 15 such questionnaires available [5]. However, there is a great deal of variability in terms of their psychometric properties and applicability across different age groups and clinical populations.

Parent-reported questionnaires are essential for the early identification of pediatric feeding issues. These tools evaluate various aspects of PE, such as the diversity of foods consumed, the presence of neophobia, and feeding-related behaviors. Some tools include direct questions about the parent’s perception (e.g., “Is your child a picky eater?”), as used by Mascola et al. [6], while others feature multi-item scales addressing complex behaviors (e.g., “My child is interested in tasting foods s/he hasn’t tasted before”) [82]. Additionally, study-specific questions are often designed for particular research cohorts. These allow for more flexibility and targeted data collection but may lack standardization, complicating data interpretation and cross-study comparisons.

Among the most extensively validated instruments is the Children’s Eating Behaviour Questionnaire (CEBQ), which evaluates several dimensions of eating behavior in children aged 2 to 10 years [82]. The “food fussiness” subscale specifically addresses PE traits, and the tool has demonstrated robust psychometric properties across diverse populations [18]. The CEBQ also provides insight into eating styles such as satiety responsiveness and food enjoyment, making it valuable for a comprehensive evaluation. It is particularly useful in distinguishing normative PE from feeding behaviors linked to emotional dysregulation or anxiety, which may require psychological intervention.

Another practical instrument is the Montreal Children’s Hospital Feeding Scale (MCH-FS), which is validated for children aged 6 months to 6 years. This questionnaire evaluates five key areas: parental concern, mealtime behavior, appetite, oral-motor function, and the parent–child feeding relationship. Each question is answered on a 7-point Likert scale. It is quick to administer and provides a global severity score that helps classify children as having typical feeding behavior, mild risk, or feeding difficulties requiring clinical intervention. The MCH-FS is particularly helpful in pediatric primary care settings where early signs of behavioral feeding issues may be subtle yetdistressing to caregivers [83].

For infants under 6 months of age, the Baby Eating Behaviour Questionnaire (BEBQ) offers insights into appetite traits and early feeding behavior [84]. It assesses enjoyment of food, responsiveness to feeding, slowness in eating, and satiety cues. Though developed for research, its application in clinical settings has grown as a tool to predict later feeding issues and to tailor advice during the introduction of solid foods.

For suspected cases of ARFID, newer instruments such as the Nine Item ARFID Screen (NIAS) are gaining clinical relevance [42]. This short, focused questionnaire targets symptoms specific to ARFID’s three main presentations: avoidance based on sensory characteristics, fear of adverse consequences like choking or vomiting, and apparent lack of interest in eating. While primarily used in specialty settings, its growing clinical relevance makes it increasingly accessible for general pediatric use.

Beyond structured questionnaires, clinical observation remains one of the most informative components of feeding assessment. Observing a child during a typical mealtime allows clinicians to evaluate oral-motor coordination, sensory responses, behavioral cues, and the interaction between the child and caregiver. A child’s reluctance to touch, smell, or taste new foods, as well as caregiver strategies—such as coercion, distraction, or reward—can be revealing. In some cases, video recordings of meals at home may offer valuable insight into the child’s natural environment and behavior.

Anthropometric measures are essential in determining the nutritional impact of feeding difficulties. Tracking weight, height, body mass index, and growth velocity over time can help identify children at risk of failure to thrive or nutritional deficiencies. The Kanawati Index, calculated by dividing mid-upper arm circumference by head circumference, is a useful screening tool in children under five years of age; values below 0.31 suggest possible undernutrition and warrant further evaluation [85]. In addition to anthropometry, a dietary history—using a 3-day or 7-day food diary—can indicate inadequate intake of macronutrients and micronutrients. When clinical signs or dietary patterns suggest deficiencies, laboratory tests may be indicated, including serum iron, ferritin, vitamin D, zinc, vitamin B12, and folate.

**Figure 1:**
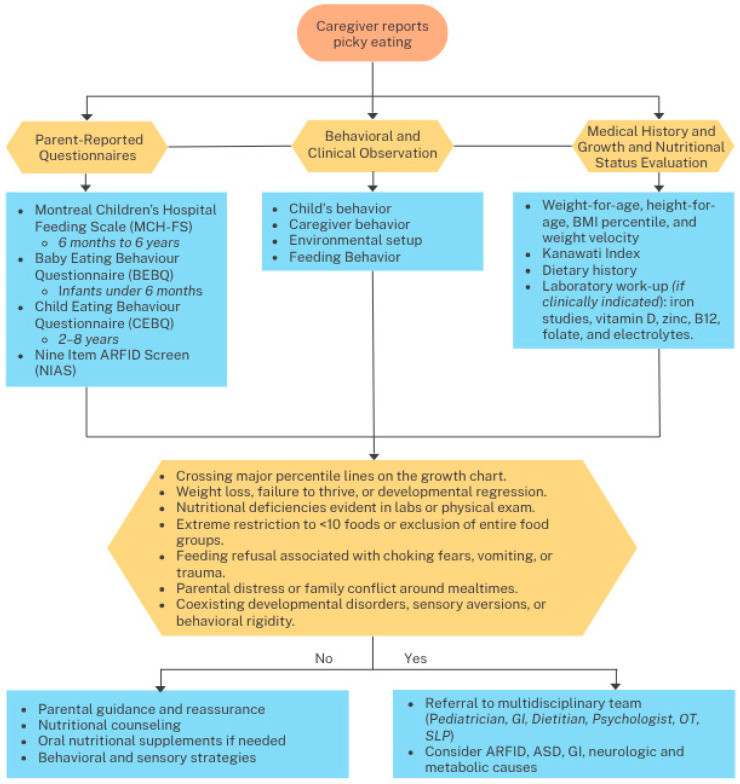
Clinical algorithm for the evaluation and management of picky eating in Children. Abbreviations: ARFID: Avoidant/Restrictive Food Intake Disorder; ASD: Autism Spectrum Disorder; GI: Gastrointestinal; OT: Occupational Therapist; SLP: Speech and Language Pathologist.

In complex cases, referral to a multidisciplinary feeding team is recommended (Figure 1). These teams typically include pediatricians, dietitians, psychologists, occupational therapists, and speech–language pathologists. Comprehensive assessment may include tools like the PARDI (Pica, ARFID, and Rumination Disorder Interview), a semi-structured interview that aids in the diagnosis of ARFID and helps differentiate it from other feeding and eating disorders [86]. The Sensory Profile 2, commonly used by occupational therapists, can assess the child’s sensory processing patterns, particularly in children with autism spectrum disorder, anxiety, or sensory sensitivities that impact feeding [87].

Specialized clinical feeding evaluations, such as those conducted by speech–language pathologists or occupational therapists, can provide detailed information on oral-motor function, texture aversions, and aspiration risk. These evaluations often form the basis of tailored feeding therapy, especially in children with significant sensory or motor-based difficulties.

Certain red flags during assessment should prompt urgent referral to a feeding specialist. These include crossing major percentile lines on the growth chart, unintentional weight loss, elimination of entire food groups, restriction to fewer than ten foods, developmental regression, and feeding refusal linked to trauma or medical complications. Significant family conflict around mealtimes or excessive parental anxiety may also indicate the need for psychological support.

In summary, assessing feeding difficulties in children requires both breadth and depth—ranging from growth charts and nutrient intake to behavioral dynamics and sensory responsiveness. By using validated tools alongside clinical expertise, pediatricians and specialists can accurately identify the nature and severity of feeding problems and guide families toward effective, individualized care.

## 9. Diagnostic Considerations and Red Flags for Organic Disease

Organic feeding disorders in children often present with persistent or severe feeding aversion, failure to thrive, or other gastrointestinal and systemic symptoms.

In a study by Chiong and al. 52% of children assesed for being selective eaters had comorbidities including autism spectrum disorder (ASD), attention-deficit/hyperactivity (ADHD) and other medical conditions such as epilepsy, eosinophilic esophagitis, previous gastroesophageal reflux disease, poor exposure to food in infancy secondary to an early onset oncologic diagnosis, and neurodegenerative disease [88].

Gastrointestinal conditions such as gastroesophageal reflux disease (GERD), eosinophilic esophagitis (EoE), and celiac disease are frequent culprits. For instance, children with GERD may exhibit chronic irritability, back arching during feeding, or frequent regurgitation [89], while EoE can manifest with vomiting, feeding refusal, and a history of atopy [90]. In celiac disease, feeding difficulties may precede more classic symptoms such as diarrhea or abdominal distension [91], particularly in younger children. Neurological conditions like cerebral palsy, hypoxic–ischemic encephalopathy, and genetic syndromes can significantly impair oral-motor coordination and swallowing ability [92]. These conditions often lead to dysphagia, aspiration risk, and dependence on alternative feeding methods. Metabolic disorders, such as phenylketonuria or maple syrup urine disease, may lead to early satiety or food refusal secondary to accumulating toxic metabolites or metabolic acidosis and often necessitate highly restrictive diets from infancy to prevent acute metabolic crises and long-term neurological damage [93].

Neurological and developmental conditions also play a significant role. Children with Structural anomalies affecting the oropharyngeal region, such as cleft palate or significant tonsillar hypertrophy, may result in mechanical difficulties with chewing and swallowing. Respiratory disorders, including chronic aspiration or congenital heart disease, can cause feeding fatigue or aversion due to the increased energy demands of respiration during eating [94].

Moreover, clinicians should remain vigilant of signs of infection or inflammation, keeping in mind potential occult/subclinical infection. Chronic infections such as Helicobacter pylori gastritis or occult urinary tract infections may result in persistent anorexia or non-specific feeding disturbances [95]. Systemic inflammatory conditions, such as inflammatory bowel disease, can cause both anorexia and nutrient malabsorption, leading to secondary feeding aversions [96,97].

In non-IgE-mediated food allergic GI disorders, most of these triggers are present in well-documented symptoms like food refusal, vomiting, and abdominal pain, starting in infancy [98].

Red flags that may indicate an organic basis for PE include failure to thrive or unexplained weight loss, persistent vomiting or dysphagia, chronic constipation or diarrhea, and signs of respiratory compromise during feeding. Other concerning features include a history of developmental delay, the presence of anemia or other micronutrient deficiencies, family history of autoimmune disease or food allergies, and evidence of malnutrition on physical examination. When such features are present, further evaluation with laboratory studies, imaging, endoscopy, or referral to a relevant specialist may be warranted.

## 10. Management Strategies

The studies on interventions for picky eaters among typically developed children found that the majority of the interventions were single-component, with the sensory approach being the most frequently utilized, followed by the nutrition approach and parenting approach. Both single and multiple intervention components improved the assessed outcomes, but the effects of other components were not evaluated in the single-component interventions [92].

According to a scoping review, occupational therapy interventions for children with autism spectrum disorder and food selectivity focused on three main categories: sensory-behavioral, family focused, and other approaches. The sensory-behavioral approach was the most commonly utilized [99].

A survey of Australian health professionals showed that common interventions for PE were education, coaching, and the sequential oral sensory approach. Occupational therapists were more likely than other allied specialties to use coaching and education [100].

### 10.1. Parental Guidance and Psychoeducation Intervention

The management of PE should begin with parental education, as parents play a central role in shaping children’s feeding behaviors. Parents often seek health professionals who listen to their concerns and provide evidence-based guidance to support healthy feeding practices [101]. Taylor and Emmett [12] outlined eight practical strategies for clinicians to share with families: (1) repeated exposure to new foods; (2) use of non-food rewards; (3) maintaining a positive, non-pressuring mealtime atmosphere; (4) role modeling by eating vegetables, fruits, and novel foods; (5) limiting snacks and high-calorie beverages between meals; (6) providing age-appropriate portion sizes; (7) eating together as a family with shared meals; and (8) focusing on consistency and long-term progress rather than short-term success. Additional parent-friendly resources are available, such as the American Academy of Pediatrics’ “10 Tips for Parents of Picky Eaters” [102]. Positive reinforcement, parental role modeling, and structured exposure therapy have all demonstrated efficacy in reducing food selectivity.

Recent findings suggest that both supportive and undermining dietary co-parenting strategies directly influence PE behaviors. Supportive strategies—such as encouraging the tasting of new foods and modeling appropriate eating behaviors—mediate positive outcomes and reduce food selectivity, while undermining practices exacerbate it [103].

Mindfulness-based approaches have also shown promise. By encouraging children to bring awareness to their sensory experience during meals, mindfulness can increase willingness to try new foods. In one study, children exposed to mindfulness techniques reported greater anticipated liking of a novel fruit and consumed more of it than controls [104].

Parent–Child Interaction Therapy (PCIT), originally designed for children aged 2–7 with behavioral difficulties, has also been applied to PE. The program improves parent–child relationships by fostering positive attention and consistent limit-setting. A recent study demonstrated that PCIT significantly reduced PE behaviors and improved caregiver feeding practices, even without specific feeding-related modules [105].

Parenting style influences feeding behaviors and practices. Midfulness may be an important mechanism through which this occurs. For example, authoritative parenting has been associated with higher levels of mindful feeding, whereas authoritarian and permissive styles are linked to decreased mindful feeding [106].

### 10.2. Nutritional Interventions

In children with limited dietary variety, nutritional supplementation may be required to address deficiencies. For example, continued use of toddler formulas enriched in iron and calcium can mitigate nutrient gaps in children over three years who avoid multiple food groups. Monitoring growth patterns and key micronutrient levels—such as iron, zinc, and vitamin D—helps guide decisions regarding supplementation [107,108].

Building on dietary counseling, randomized controlled trials (RCTs) support the judicious use of oral nutritional supplements (ONS) in children with persistent PE. In a 90-day RCT of 321 Indian preschoolers, ONS combined with dietary counseling improved micronutrient adequacy (calcium, vitamins A and C, thiamin, among others) without displacing dietary diversity, compared with counseling alone [109]. Similarly, Nogueira-de-Almeida et al. [110] demonstrated that supplementation plus guidance improved growth parameters and reduced micronutrient inadequacies among Brazilian preschoolers. A recent systematic review also found moderate evidence supporting the combination of ONS with dietetic consultation over consultation alone [111].

Consensus recommendations from the Middle East endorse a “food-first” approach—prioritizing nutrient-dense whole foods—augmented by ONS when necessary. This strategy was deemed superior to commercial fortification alone, which provided limited benefit in that region [16]. Additional evidence suggests that ONS combined with dietary counseling not only promotes catch-up growth but also reduces the incidence of upper respiratory tract infections in nutritionally vulnerable children [112].

Overall, these data indicate that ONS can safely bridge nutrient gaps and support growth in picky eaters at nutritional risk, without pathologizing what is often an adaptive developmental behavior [113].

### 10.3. Sensory Integration and Behavioral Therapy

Children with food selectivity frequently exhibit sensory sensitivities to texture, smell, temperature, or visual properties of food. Sensory Integration Therapy (SIT), widely used in occupational therapy, aims to improve sensory processing and thereby reduce feeding difficulties. Children who reject foods based on texture, temperature, smell, or appearance may benefit from systematic desensitization approaches that recalibrate sensory thresholds through gradual, positive exposures. This approach can involve a systematic hierarchy of sensory engagement, beginning with visual and olfactory exploration, progressing to tactile interaction, and culminating in oral exposure, which is reinforced through positive feedback mechanisms [114]. This approach is particularly relevant for children with autism spectrum disorder (ASD) or ARFID-like presentations. One of the most widely used sensory-behavioral interventions is the Sequential Oral Sensory (SOS) approach, which integrates elements of sensory integration theory with behavioral and cognitive strategies [115]. SOS therapy follows a hierarchy of tolerance—starting from tolerating the presence of food, to interacting with it, smelling, touching, and ultimately tasting and eating it [116]. This stepwise framework respects the child’s readiness while aiming to expand dietary repertoire without coercion.

Evidence suggests that multisensory exposure facilitates acceptance of non-preferred foods. For example, tactile play with fruits increased children’s acceptance of those foods compared to controls [117]. Similarly, Nederkoorn et al. [118] showed that preschoolers allowed to touch a novel dessert before tasting consumed significantly more than those in a control group, underscoring the importance of haptic familiarity in reducing food aversion. Visual exposure also exerts benefits: picture-book interventions featuring vegetables significantly increased willingness to taste and consume targeted foods [119,120]. Furthermore, cross-modal effects—such as container texture or food presentation—may influence taste perception: for example, smoother packaging is associated with perceived sweetness, while angular forms amplify flavor intensity, suggesting that contextual sensory cues can modulate acceptance [121].

Food chaining is another evidence-based strategy often used within sensory and behavioral therapy frameworks. This method introduces new foods with similar sensory properties to preferred items, thereby creating a gradual bridge toward broader acceptance. For example, a child who accepts chicken nuggets might be gradually exposed to homemade breaded chicken, then to roasted chicken, and eventually to other protein sources. This technique is particularly useful for children who reject entire food categories based on sensory features [122].

Behavioral interventions further enhance outcomes when combined with sensory approaches. Applied Behavior Analysis (ABA) techniques—such as shaping, positive reinforcement, and escape extinction—are effective for children with extreme selectivity, especially those with neurodevelopmental conditions. A retrospective analysis of children with feeding and eating difficulties treated with behavioral interventions at a feeding program indicated significant improvements in oral intake in 73% of participants. However, factors such as sex, the presence of a syndrome or intellectual disability, and a lack of varied nutritional intake at baseline were identified as predictors of a less favorable prognosis [123]. Parent–Child Interaction Therapy (PCIT) has also shown benefit, reducing PE behaviors through enhanced parental consistency and positive reinforcement [105].

A 2021 scoping review by Reche-Olmedo et al. identified sensory-behavioral approaches as the most frequently used interventions for children with ASD and food selectivity, followed by family-focused and other strategies. Occupational therapists were particularly likely to use coaching, education, and direct sensory desensitization to support feeding goals [99]. This multi-faceted approach underscores the critical role of interdisciplinary collaboration, often involving occupational therapists, speech–language pathologists, dietitians, and behavior analysts, to address the complex interplay of sensory, behavioral, and nutritional factors contributing to PE [99].

## 11. Prognosis and Long-Term Outcomes

Although many children gradually outgrow PE—especially with supportive interventions—a significant minority continue to exhibit selective eating well into later childhood and adolescence, raising concerns about potential nutritional and psychosocial sequelae.

The relationship between PE and weight status is inconsistent: several large cohorts report no clear link between early PE or food neophobia and subsequent BMI trajectories [63,124], yet a recent systematic review found PE to be modestly associated with underweight from birth through age 17 [125]. In a Finnish preadolescent sample (ages 9–12), children with persistent PE or food neophobia were more likely to be underweight and less likely to be overweight or obese compared to their peers [27]. Another study showed that 18% of girls remained classified as picky eaters into adolescence; yet both persistent and non-persistent groups maintained mean BMIs within the normal range as they matured [126]. Nevertheless, PE was observed less frequently, though still notably, in children with obesity. Specifically, one-third of obese children qualified as moderate picky eaters, and 17% were classified as severe picky eaters [20].

Longitudinal follow-ups offer further nuance. In the KOALA cohort, five-year-olds with and without PE exhibited similar energy intake relative to body weight [63], but higher pickiness predicted lower fruit and vegetable consumption and increased intake of sugary and trans-fatty foods over time [67,71,127,128,129]. A Chinese study also demonstrated that preschool picky eaters consumed fewer calories, protein, carbohydrates, vitamins, and minerals—and showed lower BMI-for-age—alongside biochemical evidence of magnesium, iron, and copper deficiencies [34]. Other studies confirm that micronutrient deficiencies are more common in selective eaters: zinc insufficiency affects a substantial proportion of 4–7-year-olds [108], and iron deficiency is prevalent among picky preschoolers [107].

Academic literature indicates a tendency for selective eating behaviors originating in childhood to persist into adulthood. The study from Tine et al. [130], aimed to track the long-term trajectory of selective eating and its connection to eating disorder psychopathology from birth to age 23 years. A key finding was that while 28% of the participants remained selective eaters into young adulthood, this persistence was not significantly associated with increased weight or shape concerns, interference with daily functioning, or a higher prevalence of eating disorder behaviors at age 23 [130]. Further research supports this extension into adulthood, suggesting that PE around age 4 is linked to reduced consumption of fruits, raw vegetables, cooked vegetables, and fish in early adulthood [131,132].

Beyond nutrition, persistent PE occurs alongside other developmental comcerns, in addition to various psychological and behavioral issues. School-aged picky eaters display higher rates of obsessive–compulsive symptoms and may be at increased risk of attention-deficit/hyperactivity disorder in later childhood [133]. Moderate selective eating was linked to elevated symptoms of separation anxiety and ADHD, a pattern not observed among those with severe selective eating [134]. Importantly, selective eating appears to be a marker for later psychopathology, as children with selective eating behaviors were 1.7 times more likely to exhibit increased generalized anxiety symptoms at follow-up, even after accounting for baseline levels [135]. Sensory over-responsivity appears to mediate this link: children aged 8–17 with elevated anxiety exhibit greater PE behaviors, a relationship explained by heightened sensory sensitivity [136].

These emotional and sensory challenges frequently disrupt school experiences. Children with higher levels of neophobia and pickiness exhibit lower cognitive flexibility, an executive function critical for classroom learning and problem-solving. Their working memory, inhibition, and world knowledge, however, do not appear to be directly related to their selective eating behaviors [137]. Aditionally, caregivers report that selective eaters often avoid communal mealtimes—missing lunches or refusing school-provided foods—which ontribute to social isolation and poor interaction with peers [138,139].

Together these data emphasize that persistent selective eating can extend beyond dietary restriction to impair emotional well-being, social participation, and potentially academic performance. Furthermore, these findings show that, even when weight normalizes, there can be lasting impacts on diet qualit. These findings highlight the need for early, multidisciplinary intervention.

## 12. Research Gaps and Future Directions

Advancing the understanding and management of picky eating requires coordinated, multidisciplinary efforts to close persistent gaps in diagnostic clarity, assessment methods, and intervention strategies. A key research priority is the development of standardized diagnostic frameworks that distinctly separate picky eating from overlapping conditions such as ARFID and pediatric feeding disorder. Consensus-driven criteria and validated assessment tools are essential for enhancing comparability across studies and cultural contexts, improving diagnostic precision and strengthening clinical pathways. This includes refining red-flag criteria, clarifying referral guidelines to dietitians, psychologists, speech and feeding therapists, and defining when primary care providers can manage PE.

Future research should also focus on validating sensory-based and behavioral interventions, particularly those targeting texture sensitivity, neophobia, mealtime anxiety, and rigid food preferences. Evidence supporting these approaches remains limited, and robust clinical trials are required to determine efficacy, long-term outcomes, and scalability. Parallel exploration of emerging neurobiological pathways—including sensory processing differences, neural reward systems, and neuroimmune mediators may provide insights into mechanisms that underlie selective eating and inform future therapeutic development.

Expanding the integration of digital health innovations represents another promising direction. Mobile applications, remote monitoring tools, and telehealth-based feeding therapy models may enhance accessibility and continuity of care, especially for families with limited access to specialized feeding services. Rigorous evaluation of these digital tools is necessary to determine feasibility, cost-effectiveness, and clinical utility.

Finally longitudinal studies are also required to clarify the developmental trajectory and long-term outcomes of avoidant/restrictive food intake disorder (ARFID) from childhood into adulthood.

## 13. Conclusions

PE in children is a multifaceted issue that spans typical development and complex psychopathology. While most cases are benign and transient, persistent food selectivity can impair growth, nutrition, and psychosocial functioning. A structured approach—emphasizing parental education, nutritional adequacy, behavioral strategies, and multidisciplinary collaboration—is essential for effective management. Ongoing research is vital to refine diagnostic tools and develop evidence-based interventions tailored to individual child profiles and needs.

## Figures and Tables

**Table 1:** Comparison of Picky Eating (PE), Avoidant/Restrictive Food Intake Disorder (ARFID), and Pediatric Feeding Disorder (PFD).

Feature	Picky Eating (PE)	Avoidant/Restrictive Food Intake Disorder (ARFID)	Pediatric Feeding Disorder (PFD)
Definition	Developmentally influenced selective eating with preference for limited foods	DSM-5 disorder characterized by restrictive intake due to sensory factors, fear of aversive consequences, or lack of interest	WHO 2019: Impairment in medical, nutritional, feeding skill, or psychosocial domains
Key Characteristics	Rejects familiar and unfamiliar foods; often improves with age	Restricted intake + significant weight/nutritional impairment	Feeding difficulties lasting ≥2 weeks with functional impact
Psychosocial Impact	Mild–moderate, family stress	Significant, often requiring multidisciplinary intervention	Significant; often overlaps with ARFID
Prevalence	13–50%	3–5% in pediatric population	5–25% depending on population
Red Flags	Limited variety but adequate growth	Weight loss, nutritional deficiencies, dependence on supplements	Medical instability, dysphagia, oral-motor impairment

**Table 2:** Factors Contributing to Picky Eating.

Category	Description	Impact on Feeding
Genetic	Heritability of neophobia and taste sensitivity	Shapes preference for sweet/bitter, increases selectivity
Immune/Neuroimmune	Allergy-driven aversion pathways (IL-4, IgE, GDF15)	Conditioned aversion to foods causing immune activation
Sensory Processing	Over-responsivity to taste, texture, smell	Strong aversion to non-preferred sensory properties
Temperament	Negative emotionality, shyness	Heightened refusal, more mealtime conflict
Family Environment	Pressure, restriction, parental anxiety	Reinforces maladaptive feeding patterns
Early Feeding Experiences	Tube feeding, medical trauma, delayed oral exposure	Long-term oral aversion, narrowed diet

## Data Availability

No new data were created or analyzed in this study. Data sharing is not applicable to this article.

## Abbreviations

The following abbreviations are used in this manuscript:

AAP	American Academy of Pediatrics
ABA	Applied Behavior Analysis
ADHD	Attention-Deficit/Hyperactivity Disorder
AN	Anorexia Nervosa
ARFID	Avoidant/Restrictive Food Intake Disorder
ASD	Autism Spectrum Disorder
BEBQ	Baby Eating Behaviour Questionnaire
BMI	Body Mass Index
CDC	Centers for Disease Control and Prevention
CEBQ	Children’s Eating Behaviour Questionnaire
DSM-5	Diagnostic and Statistical Manual of Mental Disorders, Fifth Edition
EoE	Eosinophilic Esophagitis
FED	Feeding and Eating Disorder
GERD	Gastroesophageal Reflux Disease
GI	Gastrointestinal
LD	Linear Dichroism (remove if not relevant; appears in template placeholder)
MCH-FS	Montreal Children’s Hospital Feeding Scale
NIAS	Nine Item ARFID Screen
ONS	Oral Nutritional Supplements
OT	Occupational Therapist
PARDI	Pica, ARFID, and Rumination Disorder Interview
PCIT	Parent–Child Interaction Therapy
PFD	Pediatric Feeding Disorder
RCT	Randomized Controlled Trial
SLP	Speech–Language Pathologist
SIT	Sensory Integration Therapy
SOS	Sequential Oral Sensory (approach)
UFED	Unspecified Feeding and Eating Disorder
WHO	World Health Organization

## References

[1] Fisher M., Zimmerman J., Bucher C., Yadlosky L.B. (2023). ARFID at 10 Years: A Review of Medical, Nutritional and Psychological Evaluation and Management. Curr. Gastroenterol. Rep..

[2] Patel M., Donovan S.M., Lee S. (2020). Considering Nature and Nurture in the Etiology and Prevention of Picky Eating: A Narrative Review. Nutrients.

[3] Dovey T.M., Staples P., Gibson E.L., Halford J.C.G. (2007). Food Neophobia and ‘Picky/Fussy’ Eating in Children: A Review. Appetite.

[4] Galloway A.T., Fiorito L.M., Lee Y., Birch L.L. (2005). Parental Pressure, Dietary Patterns, and Weight Status among Girls Who Are “Picky Eaters”. J. Am. Diet. Assoc..

[5] Taylor C.M., Wernimont S.M., Northstone K., Emmett P. (2015). Picky/Fussy Eating in Children: Review of Definitions, Assessment, Prevalence and Dietary Intakes. Appetite.

[6] Mascola A.J., Bryson S.W., Agras W.S. (2010). Picky Eating during Childhood: A Longitudinal Study to Age 11years. Eat. Behav..

[7] Ekstein S., Laniado D., Glick B.S. (2009). Does Picky Eating Affect Weight-for-Length Measurements in Young Children?. Clin. Pediatr..

[8] Carruth B.R., Ziegler P., Gordon A., Barr S.I. (2003). Prevalence of Picky Eaters among Infants and Toddlers and Their Caregivers’ Decisions about Offering a New Food. J. Am. Diet. Assoc..

[9] Brown C.L., Pesch M.H., Perrin E.M., Appugliese D.P., Miller A.L., Rosenblum K.L., Lumeng J.C. (2016). Maternal Concern for Child Undereating. Acad. Pediatr..

[10] Trofholz A., Schulte A., Berge J.M. (2016). How Parents Describe Picky Eating and Its Impact on Family Meals: A Qualitative Analysis. Appetite.

[11] Cunliffe L., Coulthard H., Williamson I. (2022). The Lived Experience of Parenting a Child with Sensory Sensitivity and Picky Eating. Matern. Child Nutr..

[12] Taylor C.M., Emmett P. (2018). Picky Eating in Children: Causes and Consequences. Proc. Nutr. Soc..

[13] Faith M.S., Heo M., Keller K., Pietrobelli A. (2013). Child Food Neophobia Is Heritable, Associated with Less Compliant Eating, and Moderates Familial Resemblance for BMI. Obesity.

[14] Tan C.C., Holub S.C. (2012). Maternal Feeding Practices Associated with Food Neophobia. Appetite.

[15] Rioux C., Lafraire J., Picard D., Blissett J. (2018). Food Rejection in Young Children: Validation of the Child Food Rejection Scale in English and Cross-Cultural Examination in the UK and France. Food Qual. Prefer..

[16] Al-Beltagi M., Choueiry E., Alahmadi N., Demerdash Z., Ayesh W., Al-Said K., Al-Haddad F., Shaaban S.Y., Tawfik E. (2024). Diet Fortification for Mild and Moderate Picky Eating in Typically Developed Children: Opinion Review of Middle East Consensus. World J. Clin. Pediatr..

[17] McCormick V., Markowitz G. (2013). Picky Eater or Feeding Disorder? Strategies for Determining the Difference. PubMed.

[18] Steinsbekk S., Bonneville-Roussy A., Fildes A., Llewellyn C., Wichstrøm L. (2017). Child and Parent Predictors of Picky Eating from Preschool to School Age. Int. J. Behav. Nutr. Phys. Act..

[19] Machado B.C., Dias P., Lima V.S., Campos J., Gonçalves S. (2016). Prevalence and Correlates of Picky Eating in Preschool-Aged Children: A Population-Based Study. Eat. Behav..

[20] Sandvik P., Ek A., Somaraki M., Hammar U., Eli K., Nowicka P. (2018). Picky Eating in Swedish Preschoolers of Different Weight Status: Application of Two New Screening Cut-Offs. Int. J. Behav. Nutr. Phys. Act..

[21] Bourne L., Bryant-Waugh R., Mandy W., Solmi F. (2023). Investigating the Prevalence and Risk Factors of Picky Eating in a Birth Cohort Study. Eat. Behav..

[22] Cole N., An R., Lee S., Donovan S.M. (2017). Correlates of Picky Eating and Food Neophobia in Young Children: A Systematic Review and Meta-Analysis. Nutr. Rev..

[23] Kozioł-Kozakowska A., Piórecka B., Schlegel-Zawadzka M. (2017). Prevalence of Food Neophobia in Pre-School Children from Southern Poland and Its Association with Eating Habits, Dietary Intake and Anthropometric Parameters: A Cross-Sectional Study. Public Health Nutr..

[24] Boyd A., Golding J., Macleod J., Lawlor D.A., Fraser A., Henderson J., Molloy L., Ness A., Ring S.M., Smith G.D. (2012). Cohort Profile: The ‘Children of the 90s’—The Index Offspring of the Avon Longitudinal Study of Parents and Children. Int. J. Epidemiol..

[25] Fernandez C., McCaffery H., Miller A.L., Kaciroti N., Lumeng J.C., Pesch M.H. (2020). Trajectories of Picky Eating in Low-Income US Children. Pediatrics.

[26] Theresa L., Mary T., Kendra H., Heather J., Rachel L., Sydney N. (2017). Picky Eating and the Associated Nutritional Consequences. J. Food Nutr. Disord..

[27] Viljakainen H., Figueiredo R.A.O., Rounge T.B., Weiderpass E. (2018). Picky Eating—A Risk Factor for Underweight in Finnish Preadolescents. Appetite.

[28] Diamantis D.V., Emmett P.M., Taylor C.M. (2023). Effect of Being a Persistent Picky Eater on Feeding Difficulties in School-Aged Children. Appetite.

[29] Moroshko I., Brennan L. (2012). Maternal Controlling Feeding Behaviours and Child Eating in Preschool-aged Children. Nutr. Diet..

[30] Goh D.Y.T., Jacob A. (2012). Perception of Picky Eating among Children in Singapore and Its Impact on Caregivers: A Questionnaire Survey. Asia Pac. Fam. Med..

[31] Chao H., Chang H. (2016). Picky Eating Behaviors Linked to Inappropriate Caregiver–Child Interaction, Caregiver Intervention, and Impaired General Development in Children. Pediatr. Neonatol..

[32] Alkazemi D., Zafar T.A., Ahmad G.J. (2025). The Association of Picky Eating among Preschoolers in Kuwait with Mothers’ Negative Attitudes and Weight Concerns. Appetite.

[33] Li Z., van der Horst K., Fries L.R., Yu K., You L., Zhang Y., Vinyes-Parès G., Wang P., Ma D., Yang X. (2016). Perceptions of Food Intake and Weight Status among Parents of Picky Eating Infants and Toddlers in China: A Cross-Sectional Study. Appetite.

[34] Xue Y., Lee E., Ning K., Zheng Y., Ma D., Gao H., Yang B., Bai Y., Wang P., Zhang Y. (2015). Prevalence of Picky Eating Behaviour in Chinese School-Age Children and Associations with Anthropometric Parameters and Intelligence Quotient. A Cross-Sectional Study. Appetite.

[35] Zubler J., Wiggins L.D., Macias M.M., Whitaker T.M., Shaw J.S., Squires J., Pajek J.A., Wolf R.B., Slaughter K., Broughton A.S. (2022). Evidence-Informed Milestones for Developmental Surveillance Tools. Pediatrics.

[36] Breiner C.E., Knedgen M.M., Proctor K.B., Zickgraf H.F. (2024). Relation between ARFID Symptomatology and Picky Eating Onset and Duration. Eat. Behav..

[37] Putnick D.L., Bell E.M., Ghassabian A., Robinson S.L., Sundaram R., Yeung E. (2021). Feeding Problems as an Indicator of Developmental Delay in Early Childhood. J. Pediatr..

[38] Lolk A. (2013). Diagnostic and Statistical Manual of Mental Disorders.

[39] Bryant-Waugh R. (2019). Feeding and Eating Disorders in Children. Psychiatr. Clin. N. Am..

[40] Hail L., Grange D.L. (2018). Bulimia Nervosa in Adolescents: Prevalence and Treatment Challenges. Adolesc. Health Med. Ther..

[41] Bertrand V., Tavolacci M., Bargiacchi A., Leblanc V., Déchelotte P., Stordeur C., Bellaïche M. (2024). Analysis of Feeding and Eating Disorders in 191 Children According to Psychiatric or Gastroenterological Recruitment: The PEDIAFED Cohort Study. Eur. Eat. Disord. Rev..

[42] Zickgraf H.F., Ellis J.M. (2017). Initial Validation of the Nine Item Avoidant/Restrictive Food Intake Disorder Screen (NIAS): A Measure of Three Restrictive Eating Patterns. Appetite.

[43] Thomas J.J., Lawson E.A., Micali N., Misra M., Deckersbach T., Eddy K.T. (2017). Avoidant/Restrictive Food Intake Disorder: A Three-Dimensional Model of Neurobiology with Implications for Etiology and Treatment. Curr. Psychiatry Rep..

[44] Kozak A., Czepczor-Bernat K., Modrzejewska J., Modrzejewska A., Matusik E., Matusik P. (2023). Avoidant/Restrictive Food Disorder (ARFID), Food Neophobia, Other Eating-Related Behaviours and Feeding Practices among Children with Autism Spectrum Disorder and in Non-Clinical Sample: A Preliminary Study. Int. J. Environ. Res. Public Health.

[45] Silvers E., Erlich K. (2023). Picky Eating or Something More? Dif Ferentiating ARFID from Typical Childhood Development. Nurse Pract..

[46] Goday P.S., Huh S.Y., Silverman A.H., Lukens C.T., Dodrill P., Cohen S.S., Delaney A.L., Feuling M.B., Noel R.J., Gisel E.G. (2018). Pediatric Feeding Disorder. J. Pediatr. Gastroenterol. Nutr..

[47] Estrem H., Park J., Thoyre S.M., McComish C., McGlothen-Bell K. (2022). Mapping the Gaps: A Scoping Review of Research on Pediatric Feeding Disorder. Clin. Nutr. ESPEN.

[48] Estrem H., Pederson J., Dodrill P., Romeo C., Thompson K., Thomas J.J., Zucker N., Noel R.J., Zickgraf H.F., Menzel J.E. (2024). A US-Based Consensus on Diagnostic Overlap and Distinction for Pediatric Feeding Disorder and Avoidant/Restrictive Food Intake Disorder. Int. J. Eat. Disord..

[49] Smith A., Herle M., Fildes A., Cooke L., Steinsbekk S., Llewellyn C. (2016). Food Fussiness and Food Neophobia Share a Common Etiology in Early Childhood. J. Child Psychol. Psychiatry.

[50] Cooke L., Haworth C.M.A., Wardle J. (2007). Genetic and Environmental Influences on Children’s Food Neophobia. Am. J. Clin. Nutr..

[51] Koenders E.A., Wesseldijk L.W., Boomsma D.I., Larsen J.K., Vink J.M. (2024). Heritability of Adult Picky Eating in the Netherlands. Appetite.

[52] Białek-Dratwa A., Szczepańska E., Szymańska D., Grajek M., Krupa-Kotara K., Kowalski O. (2022). Neophobia—A Natural Developmental Stage or Feeding Difficulties for Children?. Nutrients.

[53] Rothenberg M.E. (2023). The Immunology That Underlies Picky Eating. Nature.

[54] Plum T., Binzberger R., Thiele R., Shang F., Postrach D., Fung C., Fortea M., Stakenborg N., Wang Z., Tappe-Theodor A. (2023). Mast Cells Link Immune Sensing to Antigen-Avoidance Behaviour. Nature.

[55] Florsheim E., Bachtel N.D., Cullen J.L., Lima B.C., Godazgar M., de Carvalho F., Chatain C.P., Zimmer M.R., Zhang C., Gautier G. (2023). Immune Sensing of Food Allergens Promotes Avoidance Behaviour. Nature.

[56] Nederkoorn C., Jansen A., Havermans R.C. (2014). Feel Your Food. The Influence of Tactile Sensitivity on Picky Eating in Children. Appetite.

[57] Werthmann J., Jansen A., Havermans R.C., Nederkoorn C., Kremers S.P.J., Roefs A. (2014). Bits and Pieces. Food Texture Influences Food Acceptance in Young Children. Appetite.

[58] Bellaïche M., Leblanc V., Viala J., Jung C. (2023). Oral Exploration and Food Selectivity: A Case-Control Study Conducted in a Multidisciplinary Outpatient Setting. Front. Pediatr..

[59] Vennerød F.F.F., Nicklaus S., Lien N., Almli V.L. (2018). The Development of Basic Taste Sensitivity and Preferences in Children. Appetite.

[60] Andersen B.V., Brockhoff P.B., Hyldig G. (2018). The Importance of Liking of Appearance, -Odour, -Taste and -Texture in the Evaluation of Overall Liking. A Comparison with the Evaluation of Sensory Satisfaction. Food Qual. Prefer..

[61] Zohar A.H., Pick S., Lev-Ari L., Bachner-Melman R. (2020). A Longitudinal Study of Maternal Feeding and Children’s Picky Eating. Appetite.

[62] Zohar A.H., Lev-Ari L., Bachner-Melman R. (2019). Child and Maternal Correlates of Picky Eating in Young Children. Psychology.

[63] Antoniou E., Roefs A., Kremers S.P.J., Jansen A., Gubbels J.S., Sleddens E.F.C., Thijs C. (2015). Picky Eating and Child Weight Status Development: A Longitudinal Study. J. Hum. Nutr. Diet..

[64] Cole N., Musaad S., Lee S., Donovan S.M. (2018). Home Feeding Environment and Picky Eating Behavior in Preschool-Aged Children: A Prospective Analysis. Eat. Behav..

[65] Ellis J.M., Galloway A.T., Webb R.M., Martz D.M., Farrow C. (2015). Recollections of Pressure to Eat during Childhood, but Not Picky Eating, Predict Young Adult Eating Behavior. Appetite.

[66] Jordan A., Appugliese D.P., Miller A.L., Lumeng J.C., Rosenblum K.L., Pesch M.H. (2019). Maternal Prompting Types and Child Vegetable Intake: Exploring the Moderating Role of Picky Eating. Appetite.

[67] Kutbi H.A. (2021). Picky Eating in School-Aged Children: Sociodemographic Determinants and the Associations with Dietary Intake. Nutrients.

[68] Katzow M., Canfield C.F., Gross R.S., Messito M.J., Cates C.B., Weisleder A., Johnson S.B., Mendelsohn A.L. (2019). Maternal Depressive Symptoms and Perceived Picky Eating in a Low-Income, Primarily Hispanic Sample. J. Dev. Behav. Pediatr..

[69] Yalçın S., Oflu A., Akturfan M., Yalçın S.S. (2022). Characteristics of Picky Eater Children in Turkey: A Cross-Sectional Study. BMC Pediatr..

[70] Galloway A.T., Watson P., Pitama S., Farrow C. (2018). Socioeconomic Position and Picky Eating Behavior Predict Disparate Weight Trajectories in Infancy. Front. Endocrinol..

[71] Brown C.L., Perrin E.M., Peterson K.E., Brophy-Herb H.E., Horodynski M.A., Contreras D., Miller A.L., Appugliese D.P., Ball S., Lumeng J.C. (2017). Association of Picky Eating With Weight Status and Dietary Quality Among Low-Income Preschoolers. Acad. Pediatr..

[72] Kutbi H.A. (2020). The Relationships between Maternal Feeding Practices and Food Neophobia and Picky Eating. Int. J. Environ. Res. Public Health.

[73] Bandstra N.F., Huston P., Zvonek K., Heinz C., Piccione E. (2020). Outcomes for Feeding Tube-Dependent Children With Oral Aversion in an Intensive Interdisciplinary Treatment Program. J. Speech Lang. Hear. Res..

[74] Killian H.J., Bakula D.M., Wallisch A., Romine R.S., Fleming K., Edwards S., Bruce A.S., Chang C., Mousa H., Davis A.M. (2023). Pediatric Tube Weaning: A Meta-Analysis of Factors Contributing to Success. J. Clin. Psychol. Med. Settings.

[75] Hoffmeister J.D., Zaborek N., Thibeault S.L. (2019). Postextubation Dysphagia in Pediatric Populations: Incidence, Risk Factors, and Outcomes. J. Pediatr..

[76] Bąbik K., Patro-Gołąb B., Zalewski B., Wojtyniak K., Ostaszewski P., Horvath A. (2020). Infant Feeding Practices and Later Parent-Reported Feeding Difficulties: A Systematic Review. Nutr. Rev..

[77] Rinat S., Mackay M., Synnes A., Holsti L., Zwicker J.G. (2022). Early Feeding Behaviours of Extremely Preterm Infants Predict Neurodevelopmental Outcomes. Early Hum. Dev..

[78] Boswell N. (2021). Complementary Feeding Methods—A Review of the Benefits and Risks. Int. J. Environ. Res. Public Health.

[79] Taylor R.W., Williams S., Fangupo L.J., Wheeler B.J., Taylor B., Daniels L., Fleming E., McArthur J., Morison B., Erickson L.W. (2017). Effect of a Baby-Led Approach to Complementary Feeding on Infant Growth and Overweight. JAMA Pediatr..

[80] Białek-Dratwa A., Kowalski O. (2023). Complementary Feeding Methods, Feeding Problems, Food Neophobia, and Picky Eating among Polish Children. Children.

[81] Ortega-Ramírez A.D., Maneschy I., Miguel-Berges M.L., Pastor-Villaescusa B., Leis R., Babio N., Navas-Carretero S., Portolés O., Moreira A.S.P., Jurado-Castro J.M. (2024). Early Feeding Practices and Eating Behaviour in Preschool Children: The CORALS Cohort. Matern. Child Nutr..

[82] Wardle J., Guthrie C.A., Sanderson S.C., Rapoport L. (2001). Development of the Children’s Eating Behaviour Questionnaire. J. Child Psychol. Psychiatry.

[83] Ramsay M., Martel C., Porporino M., Zygmuntowicz C. (2011). The Montreal Children’s Hospital Feeding Scale: A Brief Bilingual Screening Tool for Identifying Feeding Problems. Paediatr. Child Health.

[84] Llewellyn C., van Jaarsveld C.H.M., Johnson L., Carnell S., Wardle J. (2011). Development and Factor Structure of the Baby Eating Behaviour Questionnaire in the Gemini Birth Cohort. Appetite.

[85] Kanawati A.A., McLaren D.S. (1970). Assessment of Marginal Malnutrition. Nature.

[86] Bryant-Waugh R., Micali N., Cooke L., Lawson E.A., Eddy K.T., Thomas J.J. (2018). Development of the Pica, ARFID, and Rumination Disorder Interview, a Multi-informant, Semi-structured Interview of Feeding Disorders across the Lifespan: A Pilot Study for Ages 10–22. Int. J. Eat. Disord..

[87] Simpson K., Adams D., Alston-Knox C., Heussler H., Keen D. (2019). Exploring the Sensory Profiles of Children on the Autism Spectrum Using the Short Sensory Profile-2 (SSP-2). J. Autism Dev. Disord..

[88] Chiong T., Tan M.L., Lim T., Quak S.H., Aw M.M. (2024). Selective Feeding—An Under-Recognised Contributor to Picky Eating. Nutrients.

[89] Rosen R., Vandenplas Y., Singendonk M., Cabana M.D., DiLorenzo C., Gottrand F., Gupta S.K., Langendam M., Staiano A., Thapar N. (2018). Pediatric Gastroesophageal Reflux Clinical Practice Guidelines. J. Pediatr. Gastroenterol. Nutr..

[90] Dias J.A., Oliva S., Παπαδοπούλου A., Thomson M., Gutiérrez-Junquera C., Kalach N., Orel R., Auth M.K., Nijenhuis-Hendriks D., Strisciuglio C. (2024). Diagnosis and Management of Eosinophilic Esophagitis in Children: An Update from the European Society for Paediatric Gastroenterology, Hepatology and Nutrition (ESPGHAN). J. Pediatr. Gastroenterol. Nutr..

[91] Catassi C., Verdú E.F., Bai J.C., Lionetti E. (2022). Coeliac Disease. Lancet.

[92] Kamarudin M.S., Shahril M.R., Haron H., Kadar M., Safii N.S., Hamzaid N.H. (2023). Interventions for Picky Eaters among Typically Developed Children—A Scoping Review. Nutrients.

[93] Camp K., Lloyd-Puryear M.A., Huntington K. (2012). Nutritional Treatment for Inborn Errors of Metabolism: Indications, Regulations, and Availability of Medical Foods and Dietary Supplements Using Phenylketonuria as an Example. Mol. Genet. Metab..

[94] Rommel N., Meyer A.D., Feenstra L., Veereman-Wauters G. (2003). The Complexity of Feeding Problems in 700 Infants and Young Children Presenting to a Tertiary Care Institution. J. Pediatr. Gastroenterol. Nutr..

[95] Homan M., Jones N., Bontems P., Carroll M., Czinn S.J., Gold B.D., Goodman K.J., Harris P., Jerris R., Kalach N. (2024). Updated Joint ESPGHAN/NASPGHAN Guidelines for Management of Helicobacter Pylori Infection in Children and Adolescents (2023). J. Pediatr. Gastroenterol. Nutr..

[96] Godny L., Dotan I. (2023). Avoiding Food Avoidance in Patients with Inflammatory Bowel Disease. United Eur. Gastroenterol. J..

[97] Bergeron F., Bouin M., D’Aoust L., Lemoyne M., Presse N. (2017). Food Avoidance in Patients with Inflammatory Bowel Disease: What, When and Who?. Clin. Nutr..

[98] Chehade M., Meyer R., Beauregard A. (2019). Feeding Difficulties in Children with Non–IgE-Mediated Food Allergic Gastrointestinal Disorders. Ann. Allergy Asthma Immunol..

[99] Reche-Olmedo L., Torres-Collado L., Compañ-Gabucio L., Hera M.G. (2021). de la The Role of Occupational Therapy in Managing Food Selectivity of Children with Autism Spectrum Disorder: A Scoping Review. Children.

[100] Chilman L.B., Meredith P., Kennedy-Behr A., Campbell G., Frakking T., Swanepoel L., Verdonck M. (2023). Picky Eating in Children: Current Clinical Trends, Practices, and Observations within the Australian Health-care Context. Aust. Occup. Ther. J..

[101] Chilman L.B., Meredith P., Southon N., Kennedy-Behr A., Frakking T., Swanepoel L., Verdonck M. (2023). A Qualitative Inquiry of Parents of Extremely Picky Eaters: Experiences, Strategies and Future Directions. Appetite.

[102] 10 Tips for Parents of Picky Eaters. https://www.healthychildren.org/English/ages-stages/toddler/nutrition/Pages/Picky-Eaters.aspx.

[103] Chen J.-L., Doong J.-Y., Tu M., Huang S.-C. (2024). Impact of Dietary Coparenting and Parenting Strategies on Picky Eating Behaviors in Young Children. Nutrients.

[104] Bennett C., Copello A., Jones C.A., Blissett J. (2020). Children Overcoming Picky Eating (COPE)—A Cluster Randomised Controlled Trial. Appetite.

[105] Peskin A., Barth A., Mansoor E., Farias A., Rothenberg W.A., Garcia D., Jent J. (2024). Impact of Parent Child Interaction Therapy on Child Eating Behaviors. Appetite.

[106] Goodman L.C., Roberts L.T., Musher-Eizenman D.R. (2019). Mindful Feeding: A Pathway between Parenting Style and Child Eating Behaviors. Eat. Behav..

[107] Saati A.A., Adly H.M. (2023). Assessing the Correlation between Blood Trace Element Concentrations, Picky Eating Habits, and Intelligence Quotient in School-Aged Children. Children.

[108] Chao H., Lu J., Yang C.-Y., Yeh P., Chu S. (2021). Serum Trace Element Levels and Their Correlation with Picky Eating Behavior, Development, and Physical Activity in Early Childhood. Nutrients.

[109] Anwar F., Yalawar M., Suryawanshi P., Ghosh A., Jog P., Khadilkar A., Kishore B., Paruchuri A.K., Pote P.D., Ravi M.D. (2023). Effect of Oral Nutritional Supplementation on Adequacy of Nutrient Intake among Picky-Eating Children at Nutritional Risk in India: A Randomized Double Blind Clinical Trial. Nutrients.

[110] Nogueira-de-Almeida C.A., Ciampo L.A.D., Martínez E.Z., Contini A.A., Nogueira-de-Almeida M.E., Ferraz I.S., Epifânio M., Ued F.d.V. (2023). Clinical Evolution of Preschool Picky Eater Children Receiving Oral Nutritional Supplementation during Six Months: A Prospective Controlled Clinical Trial. Children.

[111] Iwańska J., Pskit Ł., Stróżyk A., Horvath A., Statuch S., Szajewska H. (2025). Effect of Oral Nutritional Supplements Administration on the Management of Children with Picky Eating and Underweight: A Systematic Review and Meta-Analysis. Clin. Nutr. ESPEN.

[112] Ghosh A., Kishore B., Shaikh I.A., Satyavrat V., Kumar A., Shah T., Pote P., Shinde S., Berde Y., Low Y.L. (2018). Effect of Oral Nutritional Supplementation on Growth and Recurrent Upper Respiratory Tract Infections in Picky Eating Children at Nutritional Risk: A Randomized, Controlled Trial. J. Int. Med. Res..

[113] Veronica S.-W.M. (2016). How Picky Eating Becomes an Illness—Marketing Nutrient-Enriched Formula Milk in a Chinese Society. Ecol. Food Nutr..

[114] Maggio R., Turriziani L., Suraniti S., Graziano M., Patanè S., Randazzo A., Passantino C., Cara M.D., Quartarone A., Cucinotta F. (2024). Case Report: Multicomponent Intervention for Severe Food Selectivity in Autism Spectrum Disorder: A Single Case Study. Front. Psychiatry.

[115] Kim A.-R., Kwon J., Yi S., Kim E. (2021). Sensory Based Feeding Intervention for Toddlers With Food Refusal: A Randomized Controlled Trial. Ann. Rehabil. Med..

[116] Schoen S., Balderrama R., Dopheide E., Harris A., Hoffman L., Sasse S. (2025). Methodological Components for Evaluating Intervention Effectiveness of SOS Feeding Approach: A Feasibility Study. Children.

[117] Coulthard H., Williamson I., Palfreyman Z., Lyttle S. (2017). Evaluation of a Pilot Sensory Play Intervention to Increase Fruit Acceptance in Preschool Children. Appetite.

[118] Nederkoorn C., Theiβen J., Tummers M., Roefs A. (2017). Taste the Feeling or Feel the Tasting: Tactile Exposure to Food Texture Promotes Food Acceptance. Appetite.

[119] Owen L., Kennedy O., Hill C., Houston-Price C. (2018). Peas, Please! Food Familiarization through Picture Books Helps Parents Introduce Vegetables into Preschoolers’ Diets. Appetite.

[120] Rioux C., Lafraire J., Picard D. (2017). Visual Exposure and Categorization Performance Positively Influence 3- to 6-Year-Old Children’s Willingness to Taste Unfamiliar Vegetables. Appetite.

[121] van Rompay T.J.L., Kramer L.-M., Saakes D. (2018). The Sweetest Punch: Effects of 3D-Printed Surface Textures and Graphic Design on Ice-Cream Evaluation. Food Qual. Prefer..

[122] Fishbein M., Cox S., Swenny C., Mogren C., Walbert L., Fraker C. (2006). Food Chaining: A Systematic Approach for the Treatment of Children With Feeding Aversion. Nutr. Clin. Pract..

[123] Dumont É., Jansen A., Duker P.C., Seys D.M., Broers N.J., Mulkens S. (2023). Feeding/Eating Problems in Children: Who Does (Not) Benefit after Behavior Therapy? A Retrospective Chart Review. Front. Pediatr..

[124] Brown C.L., Schaaf E.B.V., Cohen G.M., Irby M.B., Skelton J.A. (2016). Association of Picky Eating and Food Neophobia with Weight: A Systematic Review. Child. Obes..

[125] Jani R., Irwin C., Rigby R.R., Byrne R., Love P., Khan F., Larach C., Yang W.Y., Mandalika S., Knight-Agarwal C.R. (2024). Association Between Picky Eating, Weight Status, Vegetable, and Fruit Intake in Children and Adolescents: Systematic Review and Meta-Analysis. Child. Obes..

[126] Berger P.K., Hohman E.E., Marini M.E., Savage J.S., Birch L.L. (2016). Girls’ Picky Eating in Childhood Is Associated with Normal Weight Status from Ages 5 to 15 y. Am. J. Clin. Nutr..

[127] Taylor C.M., Northstone K., Wernimont S.M., Emmett P. (2016). Macro- and Micronutrient Intakes in Picky Eaters: A Cause for Concern?. Am. J. Clin. Nutr..

[128] Sandvik P., Ek A., Eli K., Somaraki M., Bottai M., Nowicka P. (2019). Picky Eating in an Obesity Intervention for Preschool-Aged Children—What Role Does It Play, and Does the Measurement Instrument Matter?. Int. J. Behav. Nutr. Phys. Act..

[129] van der Horst K., Deming D.M., Lesniauskas R., Carr B.T., Reidy K. (2016). Picky Eating: Associations with Child Eating Characteristics and Food Intake. Appetite.

[130] Tine M.L.V., McNicholas F., Safer D.L., Agras W.S. (2017). Follow-up of Selective Eaters from Childhood to Adulthood. Eat. Behav..

[131] Pesch M.H., Bauer K.W., Christoph M.J., Larson N., Neumark-Sztainer D. (2019). Young Adult Nutrition and Weight Correlates of Picky Eating during Childhood. Public Health Nutr..

[132] Pereboom J., Thijs C., Eussen S.J.P.M., Mommers M., Gubbels J.S. (2023). Association of Picky Eating around Age 4 with Dietary Intake and Weight Status in Early Adulthood: A 14-Year Follow-up Based on the KOALA Birth Cohort Study. Appetite.

[133] Schwarzlose R.F., Hennefield L., Hoyniak C.P., Luby J.L., Gilbert K. (2022). Picky Eating in Childhood: Associations With Obsessive-Compulsive Symptoms. J. Pediatr. Psychol..

[134] Wolstenholme H., Kelly C., Hennessy M., Heary C. (2020). Childhood Fussy/Picky Eating Behaviours: A Systematic Review and Synthesis of Qualitative Studies. Int. J. Behav. Nutr. Phys. Act..

[135] Zucker N., Copeland W., Franz L., Carpenter K.L.H., Keeling L.A., Angold A., Egger H.L. (2015). Psychological and Psychosocial Impairment in Preschoolers With Selective Eating. Pediatrics.

[136] Zickgraf H.F., Elkins A.R. (2018). Sensory Sensitivity Mediates the Relationship between Anxiety and Picky Eating in Children/ Adolescents Ages 8–17, and in College Undergraduates: A Replication and Age-Upward Extension. Appetite.

[137] Foinant D., Lafraire J., Thibaut J. (2022). Relationships between Executive Functions and Food Rejection Dispositions in Young Children. Appetite.

[138] Gent V., Marshall J., Weir K.A., Trembath D. (2025). Caregiver Perspectives Regarding the Impact of Feeding Difficulties on Mealtime Participation for Primary School-Aged Autistic Children and Their Families. Int. J. Speech-Lang. Pathol..

[139] Chapman L.E., Gosliner W., Olarte D.A., Zuercher M.D., Ritchie L.D., Orta-Aleman D., Schwartz M.B., Polacsek M., Hecht C.E., Hecht K. (2025). Impact of Mealtime Social Experiences on Student Consumption of Meals at School: A Qualitative Analysis of Caregiver Perspectives. Public Health Nutr..

